# Soil pH and Soluble Organic Matter Shifts Exerted by Heating Affect Microbial Response

**DOI:** 10.3390/ijerph192315751

**Published:** 2022-11-26

**Authors:** Gael Bárcenas-Moreno, Elizabeth Jiménez-Compán, Layla M. San Emeterio, Nicasio T. Jiménez-Morillo, José A. González-Pérez

**Affiliations:** 1 MED Soil Research Group, Departmento de Cristalografía, Mineralogía y Química Agrícola, Facultad de Química, Universidad de Sevilla, C/Prof Garcia Gonzalez 1, 41012 Sevilla, Spain; 2 Instituto de Recursos Naturales y Agrobiología de Sevilla, Consejo Superior de Investigaciones Científicas (IRNAS-CSIC), Av. Reina Mercedes 10, 41012 Sevilla, Spain; 3 Instituto Mediterrâneo para a Agricultura, Ambiente e Desenvolvimento (MED), University of Évora, Núcleo da Mitra, Ap. 94, 7006-554 Évora, Portugal

**Keywords:** laboratory heating, soil pH, soil organic matter (SOM), pyrolysis–gas chromatography–mass spectrometry (Py-GC/MS), soil micro-organisms, colony forming units (CFU)

## Abstract

Fire-induced alterations to soil pH and organic matter play an important role in the post-fire microbial response. However, the magnitude of which each parameter affects this response is still unclear. The main objective of this work was to determine the magnitude in which soil pH and organic matter fire-induced alterations condition the response of viable and cultivable micro-organisms using laboratory heating, mimicking a range of fire intensities. Four heating treatments were applied to unaltered forest soil: unheated, 300, 450, and 500 °C. In order to isolate the effect of nutrient or pH heating-induced changes, different culture media were prepared using soil:water extracts from the different heated soils, nutrient, and pH amendments. Each medium was inoculated with different dilutions of a microbial suspension from the same original, unaltered soil, and microbial abundance was estimated. Concurrently, freeze-dry aliquots from each soil:water extract were analyzed by pyrolysis-gas chromatography/mass spectrometry. The microbial abundance in media prepared with heated soil was lower than that in media prepared with unheated soil. Nutrient addition and pH compensation appear to promote microbial proliferation in unaltered and low-intensity heated treatments, but not in those heated at the highest temperatures. Soil organic matter characterization showed a reduction in the number of organic compounds in soil-heated treatments and a marked increase in aromatic compounds, which could be related to the observed low microbial proliferation.

## 1. Introduction

Fire has become a major threat to nature, especially in the last few decades when the incidence of big fires and megafires has increased. Despite the decrease in the number of fire events, mainly due to the improvement of fire detection and the extinguishing media, the total burned area follows a positive trend worldwide, destroying immense forested areas of incalculable ecological value. Between 2009 and 2018, the northern Mediterranean area had an average of more than 56,000 fire incidents, with c. 375,000 h burned every year [1]. During the last 20 years, fires incidents have been especially critical for some countries in western Europe, for example, just accounting fires in 2005, 2012, and 2017, more than half a million hectares burned in Spain. This phenomenon is not limited to the south of Europe, but northern and central European areas have also experienced important fires due to climate change. The increased frequency of unusual dry summers in these regions has recently caused large fires in countries, such as Sweden, Germany, Poland, and the United Kingdom [1,2].

In Mediterranean areas, many plants have evolved with fire, so forest vegetation usually includes several species well adapted to or even dependent on fire [3,4]. Nevertheless, soils are one of the most susceptible nonrenewable resources, prone to the effects of fire. Wildfires not only destroy the plant cover but also alter soil properties, such as soil organic matter [5,6] or aggregate stability [7]. This usually leads to unprotected soil and increased soil erosion risks.

Fire effects on soils and ecosystems are conditioned mainly by fire severity, which can be estimated by soil organic matter (SOM) destruction [4]. The loss and transformation of soil organic compounds, fire-induced pH changes, or the impact on physical parameters, such as water, repellence, or aggregate stability [8,9,10,11], are some of the most frequent fire effects that could delay ecosystem recovery.

Soil micro-organisms are also affected by fire, both directly and indirectly [12]. This will largely depend on the temperatures the soil reaches during burning, which acts as the first filter for selecting surviving micro-organisms [13]. Soil heated above 200 °C is usually enough to sterilize the soil [14], but heat transmission through the soil usually limits lethal temperatures mostly up to the first 5 cm of soil [15]. After this first effect of the temperature, the surviving micro-organisms will face other constraints, such as indirect effects caused by soil conditions, which can promote or inhibit different members of the original microbial community, altering its composition. 

The heterogeneous impact of fire, together with the variability derived from the methodology selected to evaluate fire effects on soil micro-organisms, has provided contradictory results that make it difficult to discern key factors controlling microbial responses to fire [13]. Fire can alter basic factors controlling microbial proliferation, such as water content, pH, or nutrient availability [6,11], but sometimes, changes caused by fire in some parameters could result in stimulating microbial proliferation and, at the same time, may affect other parameters that promote an inhibitory effect. For example, depending on the original soil characteristics, soil pH can increase, decrease, or stay as unheated samples. Increased pH values due to fire are the most extended effect and are usually related to the liming effects of ashes [6,16]. This increase in soil pH after a fire has been usually related to bacterial proliferation above fungi after fire [9,14,17], either due to bacteria favored by the high pH [18] or due to better rates of survival of bacteria compared to fungi favoring larger initial bacterial inoculum [17]. Nevertheless, any pH deviation from the original condition, could result in an important factor in the recolonization process, since the adaptation to the new media could not be the same for all the components of microbial communities, and may even vary depending on the direction of the change [19].

On the other hand, fire-induced alteration of soil organic matter (SOM) can also condition microbial responses in different ways. At low-moderate severity fires, SOM content can slightly decrease or even increase due to the incorporation of charred plant material, whereas higher fire severity usually results in a marked loss of SOM [5,6,11]. The literature presented a variety of results that were contradictory, evidencing that the effect of SOM changes on soil micro-organisms, not only depend on the type of fire-induced change, but also on the microbial parameters studied [9,14]. Other than the alteration of SOM content, we should take into account, changes in SOM availability. Increases in dissolved organic carbon (DOC) have been reported in numerous studies and are associated with low-moderate severity fires [9,14,17,20]. Soil respiration rate dynamics after fire have been previously correlated to chronological DOC changes [9,14,17,21], and also, marked bacterial proliferation has been previously related to the combined effect of increases in DOC and pH values [9,17,21,22]. The content and availability of SOM cannot always entirely explain the microbial response after fire, since qualitative SOM changes can be also decisive in the colonization process. The alteration of different organic fractions depending on the temperatures reached, and the formation of pyromorphic material characterized by low solubility, high aromaticity, and recalcitrance, presumably will condition microbial proliferation. Previous studies have reported negative effects on soil micro-organisms due to fire-induced compounds present in soil:water extracts, such as in fungi [23] and bacteria [24]. Nevertheless, the dynamic and duration of these compounds in the soil and their effects on the microbial population are still unclear, due to the lack of studies that include the monitoring of fire effects on both, microbial parameter responses and quality changes in soil organic matter. 

Therefore, enlightening the effect of soil critical factors on post-fire microbial response depending on fire severity could lead to relevant advances, not only for a better understanding of the soil physicochemical parameters’ dynamics after the fire but also in helping decision-makers in the design of appropriate post-fire management strategies.

The main objective of this work is to elucidate how heat-induced soil pH and organic matter shifts condition the response of micro-organisms after the fire. For this, a laboratory heating experiment was designed to mimic different fire intensities intended to (1) determine to what extent soil:water extracts affect soil micro-organisms (2) to estimate how this conditions microbial response and monitor its dynamic (3) to study heating-induced quality changes on soil organic matter as a determinant factor in microbial response.

In spite of the fact that this experiment will not reflect all possible natural conditions or all the components of the microbial population, we hypothesize that this design will allow us to detect and characterize some variables of this complex equation in a simple way and lead us to elucidate key questions that may serve as keystones for setting out future studies.

## 2. Materials and Methods

### 2.1. Soil Samples

The soils studied were collected in Nigüelas (Granada, Spain 3°28′46.21″ W 36°59′49.56″ N), an area of high ecological value under high-mountain vegetation in Sierra Nevada National Park at 2000 m above sea level. The area was affected by an important wildfire in 2006 [25]. The characteristic vegetation of this area is known as “Piornal” usually growing between 1800–3100 m.a.s.l. where *Genista versicolor* Boiss, *Cytisus oromediterraneous* (Boiss.) Rivas Martínez and *Berberis hispanica* Boiss. and Reut. are dominant species. The mean annual temperature in the nearest town (Lanjaron at 710 m.a.s.l.) is 14.7 °C, and the annual mean precipitation is 513 mm. Nevertheless, lower mean temperature (8–13 °C) and quantitative and qualitative changes in the annual precipitations are expected due to the higher elevation in the sampling area, reaching annual precipitation higher than 800 mm with abundant snowfalls [26]. The soil type is a cryochrept on a siliceous substrate as the parent material, with a sandy loam texture, according to the USDA Soil Taxonomy [27].

Soil samples were collected in transects, and a composed soil sample were formed mixing four subsamples from the 5 first cm of soil after litter removal. The samples were kept at 4 °C and immediately transported to the laboratory where samples were air-dried and sieved (<2 mm) before the soil characterization and heating. 

### 2.2. Experimental Design

Soil from the unaltered area described above was heated in a muffle furnace at different temperatures to reach a range of fire intensities: unheated-control soil (UH) and soil heated at 300, 450, and 500 °C during 20 min. After heating, the samples were cooled under sterile conditions and stored at 4 °C until used. The soil samples from the different soil heating treatments were used to prepare culture media with soil:water extracts. In order to control the influence of nutrient and pH alteration induced by the heating process, nutrient supplements and pH correction were applied, obtaining 11 different cultures as described in Table 1. Each culture medium was inoculated with a different dilution of the same microbial suspension prepared from the original, unaltered soil. 

### 2.3. Soil Characterization

Soil texture and particle size distribution were measured using a Bouyoucos hydrometer [28]. The water-holding capacity (WHC) was determined according to Foster [29]. The soil water content (SWC) was estimated by drying at 105 °C overnight, and the soil pH and electrical conductivity (EC) were measured in deionized water (1:2 and 1:5 w:w, respectively) at 20 °C. The soil organic C (SOC) was analyzed using rapid dichromate oxidation of organic C [30], and the dissolved organic C (DOC) was measured in soil:water extract used to prepare culture media as in non-fumigated soil samples, as reported for microbial biomass C [31]. The soil nitrogen (Nk) was estimated by Kjeldahl digestion [32]. Microbial biomass, respiration rate, leu-incorporation and fungal and bacterial PLFAs were also determined as described in Bárcenas-Moreno et al. [25].

### 2.4. Organic Matter Analysis Composition Measured by Analytical Pyrolysis (Py-GC/MS)

Direct pyrolysis–gas chromatography–mass spectrometry (Py-GC/MS) analysis was performed as in Jiménez-Morillo et al. [33] using a double-shot pyrolyser (model 2020i; Frontier Laboratories Ltd., Fukushima, Japan) attached to a GC/MS system the Agilent 6890N/5973MSD (Agilent Technologies, Santa Clara, CA, USA). An aliquot of 3 mL from the same soil: water extract used to prepare the culture media were freeze-dried and placed in small, deactivated stainless-steel capsules, introduced into a preheated micro-furnace at 500 °C for 1 min, and the evolved gases were transferred into the GC/MS for analysis. The gas chromatograph was equipped with a capillary column (Agilent J and WHP-5 ms UI) of 30 m × 250 μm × 0.25 μm film thickness. The oven temperature was held at 50 °C for 1 min and then increased to 100 °C at 30 °C min^−1^, from 100 °C to 300 °C at 10 °C min^−1^, and stabilized at 300 °C for 10 min using a heating rate of 20 °C min^−1^ in the scan mode. The carrier gas used was He at a controlled flowrate of 1 mL min^−1^. The mass spectra were acquired at 70 eV ionizing energy. Compound assignment was achieved via single-ion monitoring for various homologous series, utilizing low-resolution mass spectrometry. Results were compared with published and stored (NIST and Wiley libraries) data. The relative abundance of each pyrolysis product was calculated as a percentage of the chromatographic area of the identified compounds. Compounds were identified and quantified only when they reached a relative area greater than 0.25% of the total area of the total ion chromatogram (TIC).

In order to visualize more accurately the tendency in organic compounds transformation induced with increasing heating temperatures, Van Krevelen diagrams were elaborated using H/C and O/C atomic ratios as calculated from the molecular formula of the identified pyrolysis products.

### 2.5. Microbial Abundance Estimation

The abundance of viable and cultivable micro-organisms was estimated by the plate count method following Zuberer [34]. It is known that the number of micro-organisms isolated by this medium is underestimated and represents only a small percentage of the total micro-organisms growing in soil. However, it is considered a useful tool for comparative studies, such as this one, where the effects of different heating treatments are compared on the same portion of the microbial population growing on culture media made with soil:water extracts.

### 2.6. Culture Media Preparation

Eleven culture media were created based on soil:water extracts as described in Table 1. Soil and water were mixed in 1:2 (w:w) proportion and shaken during 2 h [24]. Then the extract was filtered to discard soil particles and used to prepare different culture media.

Before the preparation of culture media, an aliquot of each soil:water extract was taken to measure pH, DOC and organic matter. Then, the extracts were mixed with deionized water or nutritive solution (N^+^) depending on the treatment (1:1, v:v). Bacteriological agar was added at 15% to solidify the medium. The mix was then autoclaved at 121 °C for 15 min before inoculation with the microbial suspension. In order to visualize possible autoclaving influence on soluble organic composition of the media, an aliquot of unheated soil treatment was autoclaved under the same conditions and also analyzed with Py-GC/MS following the same steps described before.

The amount of nutrient added in the N+-treatments was performed following the indications to prepare “soil extract Agar” as described in Wollum [35], using glucose (1 g L^−1^) to obtain at least 0.4 g C L^−1^, yeast extract (5 g L^−1^) and K_2_HPO_4_ (0.2 g L^−1^) as sources of N and P.

The treatment N^+^-pH, was prepared as described previously for N^+^-treatment but the pH was adjusted to pH 6, that was close to the original soil pH before the heating treatments.

In addition, N^+^ Agar (1 g L^−1^ of glucose, 5 g L^−1^ of yeast extract, 0.2 g L^−1^ of K_2_HPO_4_ and 15 g L^−1^ of bacteriological agar) was used as a control and to check the maximal microbial growth that could support the amount of nutrient added to the media.

Tryptic Soy Agar (TSA), frequently used to isolate soil heterotrophic micro-organisms, was also prepared as a reference of culture medium.

### 2.7. Plates Inoculation and Spreading

To activate the maximum amount of micro-organisms present in the soil, dry and sieved soil samples were rewetted to reach 50–60% WHC 4 days before the inoculation of the medium. This soil was used to prepare a serial dilution, and 0.1 mL of different dilutions were inoculated and spread in all the media. The incubation temperature was 25 °C and colony forming units (CFU) were quantified during 5 days of incubation, since micro-organisms in the media with heated soil and without nutrient addition take more time to grow. The treatments were performed in triplicate.

### 2.8. Statistical Analyses

Normal distribution and variance homogeneity were tested for all samples using the Kolmogorov–Smirnov and Levene tests, respectively. Colony forming unit (CFU) data were necessary transformed using log10 (x + 1). The possible changes in microbial abundance due to the different treatments were analyzed by one-way ANOVA and a Tukey’s post-hoc test using the IBM SPSS Statistic package. Differences were considered statistically significant at *p* < 0.05.

In order to clarify the possible existence of different organic matter transformations at each heating treatment, the relative abundance of the organic compounds identified by Py-GC/MS were analyzed by principal component analysis (PCA) using the MVSP 3.0 package.

## 3. Results and Discussion

### 3.1. Soil Characterization

The main physical and chemical characteristics of unaltered soil are shown in Table 2, as well as microbial parameters.

### 3.2. Characterization of Soil: Water Extract to Culture Media

**Figure 1:**
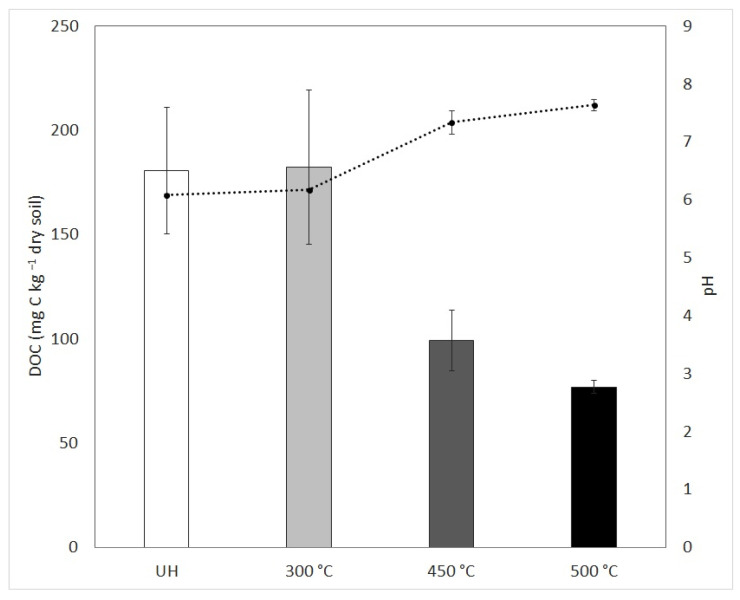
Mean value (±standard error) of DOC (dissolved organic carbon mg C kg^−1^ dry soil) (bars) at left axis and pH (dotted line) at right axis measured in soil:water extract 1:4 and 1:2, respectively.

In order to establish carbon availability to microbial growth in the culture media, dissolved organic carbon (DOC) and pH were analyzed after laboratory heating, taking an aliquot from the soil:water extract prepared to make culture media just before autoclaving. Figure 1 shows DOC and pH before some compensation was made to prepare N^+^ and N^+^-pH treatments. Soil laboratory heating above 300 °C for 20 min induced a conspicuous DOC destruction, this diminution appears to be proportional to the heating temperature. Organic matter combustion and consequent carbon losses appear between 220 and 460 °C [36,37] although it is possible to find considerable DOC rises at samples heated between 200–300 °C in laboratory experiments [14] or even as a short-term effect on fire-affected areas [9,38,39]. On the contrary to this DOC tendency, the pH of the extract increase with heating temperature. The pH increase has been related to organic acid denaturation [40]. In line with this, the analytical pyrolysis results evidence the disappearance of benzoic acid in all the heated soil extracts, but the pH rise did not show the same magnitude in all the heated treatments. According to Certini [16], significant increases in soil pH after fire only occur at high temperatures (>450 °C) due to the total fuel combustion and consequent base release, but other authors related this phenomenon to the loss of hydroxyl groups in clays [41] or the amount of iron and aluminum oxides [42,43]. We should take into account that the pH was measured in the soil:water extract before the preparation of the different culture media, so the final pH after adding nutrients could slightly alter pH in those treatments where pH was not restored, making it more neutral in both, unheated and heated conditions, clouding the real pH fire-induced effect.

### 3.3. Heating Effect on Organic Matter Composition

**Figure 2:**
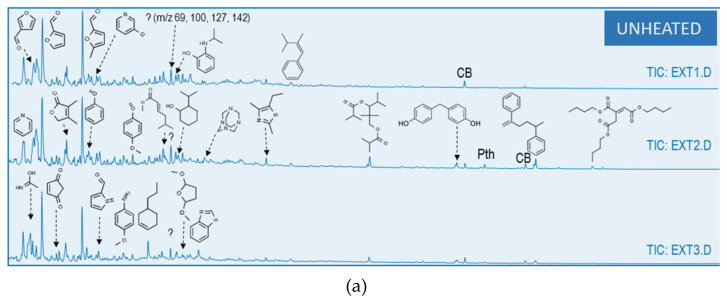

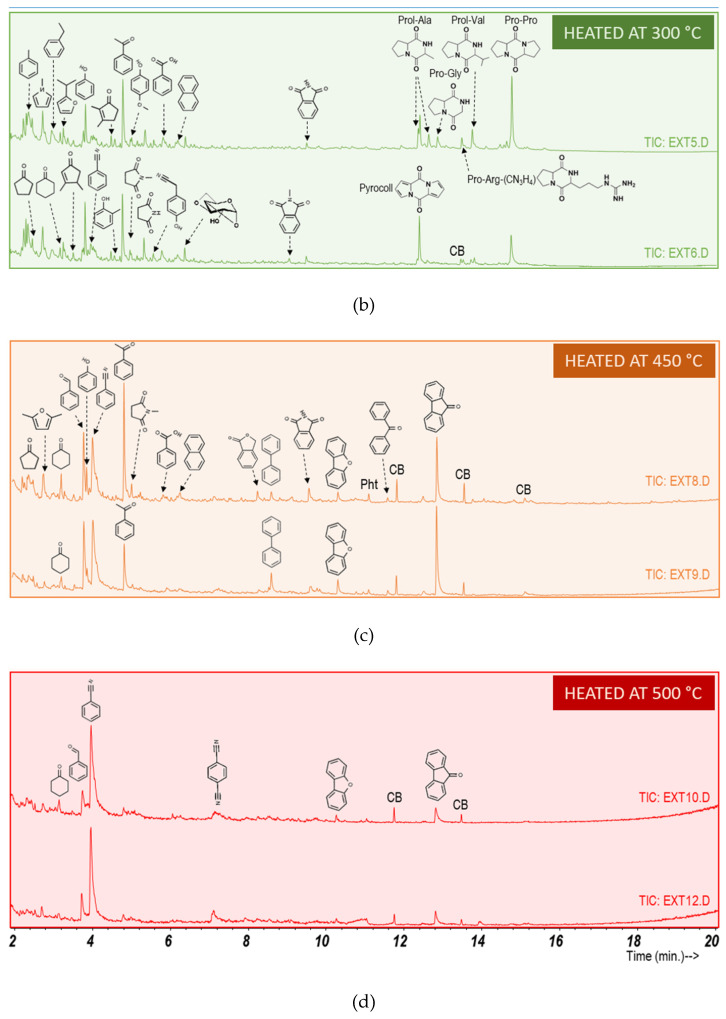
Total-ion current (TIC) chromatogram obtained from GC-MS analysis of the soil:water extract used to prepare different culture medias. (**a**) Unheated-control; (**b**) heated at 300 °C; (**c**) heated at 450 °C and (**d**) heated at 500 °C. The main identified compounds have been represented.

The pyrograms obtained from the soil:water extracts were well resolved and a total of 68 different compounds were identified (Table 3, Figure 2). At first sight, a clear decrease in the number of peaks is observed as the temperature of heating increases. The distribution of the major peaks along the pyrograms changes as well, from small molecules mainly distributed in the first 6 min of the chromatogram for the unheated soil:water extract, to larger compounds (RT: 4–15 min) in the heated samples. This change in the distribution of the peaks can be attributed to the formation of organic compounds with higher molecular weight in the heated samples that includes aromatic (benzaldehyde, dibenzofuran or fluorenone) and N compounds (benzonitrile or dicyanobenzene) and the destruction of the most thermolabile molecules (polysaccharides and polypeptides) and other low molecular weight compounds from plant biomass [33] which were present in the pyrogram of unheated samples. For this reason, the pyrograms of soil:water extract heated at 300 or even 450 °C showed higher relative abundance of compounds such as toluene (RT = 2.39 min), phenol (Rt = 3.85 min) and acetophenone (RT = 4.80 min), that are attributed to the pyrolysis of lignin and cellulose [44], as well as the appearance of compounds in the highest retention times that did not appear in the control, such as pyrocol and a number of diketopiperazines from proteins [45,46]. In the pyrograms at 450 and 500 °C, the diversity of compound s is much lower and are more defined.

**Figure 3:**
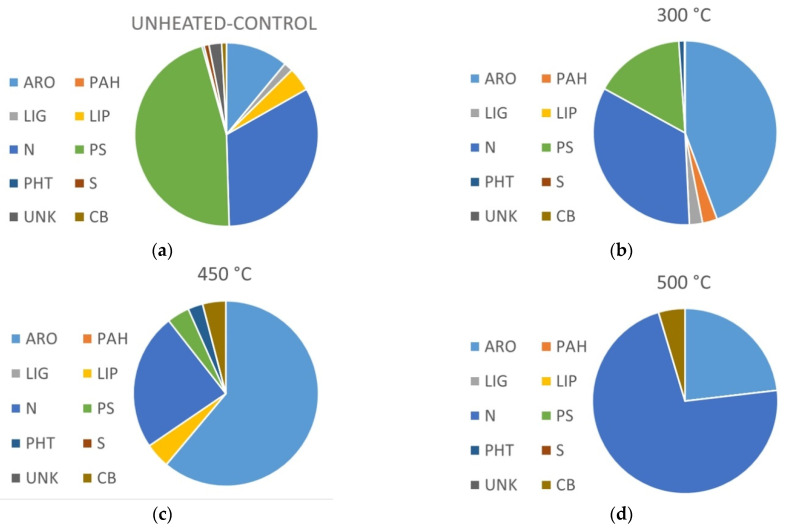
Composition of the pyrolysates of unheated control (**a**), heated at 300 (**b**), 450 (**c**), and 500 °C (**d**) with an indication of their probable origin: ARO: aromatic and hydroaromatic; PAH: polycyclic aromatic hydrocarbons; LIG: lignin derivatives; LIP: lipids; N: nitrogenous; PHT: phthalates (contaminant); S: sulfur; UNK: unknown; and CB: column bleed.

To synthesize the information, the compounds identified have been grouped according to their origin. Figure 3 shows the relative abundance of aromatic and hydroaromatic (ARO), polycyclic aromatic hydrocarbons (PAH), polysaccharides (PS), derivatives of lignin (LIG), lipids (LIP), nitrogenous (N), phthalates (PHT pollutant) and sulfur (S). The part corresponding to unknown compounds (UNK) and column bleed (CB) are also identified in the graphs. A higher amount of PS is found in the unheated samples compared with the heating ones. Samples heated at 300 °C, evidence an increase in the relative abundance of ARO compounds compared to the control. The most severe heating treatments showed lower number of peaks. In the pyrogram of samples heated at 450 °C the ARO compounds predominate as well as compounds with nitrogen (N), while the pyrogram from 500 °C heated samples was the poorer in peak diversity (6 peaks) and dominated by nitrogen-bearing aromatic molecules. It is worth highlighting that the samples burned at 300 °C harbour both, fire-related substances such as polycyclic aromatic hydrocarbons (PAH) as well as molecules derived directly from plant biopolymers, such as lignin, cellulose and polypeptides or proteins.

**Figure 4:**
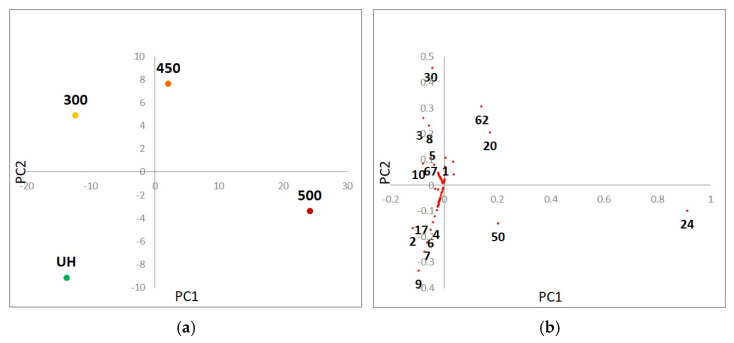
The principal component analysis (PCA) scores plot (**a**) and the corresponding PCA loadings plot (**b**) of the multivariate statistical analysis of the relative abundance of different compounds identified from the soil:water extracts by Pyr-GC/MS. Those compounds significantly related to the separation of different samples have been marked (the numbers refer to the compounds (Cmp) in Table 3).

In order to reveal the main changes in organic matter quality induced by heating, a Principal Component Analysis (PCA) was performed. Figure 4 shows the loading and scores of the multivariate statistical analysis of the relative abundance of different compounds identified from the soil:water extracts by Pyr-GC/MS. A 77.6% of the variability is explained by PC 1 sorting the treatment along the *x* axis, from the unheated-control (UH) and low intensity heating (300 °C) in the negative values to the higher heating temperature (450–500 °C) in the positive ones. The PC2 accounted for 14.7% of the variability and seems to discriminate markedly samples heated at 300 and 450 °C, with positive values from the UH with negative values. The compounds are found also significantly related with this distribution of the samples in the PCA. Benzonitrile (Table 3: Cmp. **24**) is the only compound related solely to PC1, the compounds (Cmp). **2**, **20**, **50**, **62** are significantly related to both PCs, while Cmps. **1**, **3**–**10**, **17**, **19**, **30**, **67** were significantly related to PC2.

The pyrolysis of UH samples reveal polysaccharides as dominant composition, followed by N-compounds, although aromatic compounds, lignin derivatives and lipids were also detected. All these compounds are typically synthesized by living organisms and have been the predominant compounds detected in the TIC of the control sample of other Mediterranean forest [47]. The higher peaks in UH samples were related to furfural (Cmps. **7**, **9**), a natural dehydration product of xylose, a monosaccharide often found in large quantities in the hemicellulose fraction of lignocellulosic biomass, from which it is almost exclusively produced [48]. Furfural has been lately studied for its potential use for the synthesis of chemicals and fuels based on lignocellulosic biomass of different plant species, mainly grasses [49,50]. The study area where soil samples were collected was characterised by high mountain shrubs accompanied by grasses as Festuca indigesta Boiss. which could be an important source of furfural [51]. The compound 2(5H)-Furanone (Cmp. **17**) was also found in UH samples. Natural production of this compound has been attributed to some actinobacteria as Streptomyces sp. and even to some fungal species such as *Favolaschia* sp. and *Fusarium* sp. [52]. Pyridine (2) was significantly related to PC1 and PC2, and is present in UH and 300 °C treatments. Pyridine can be present in the environment from the breakdown of different natural materials [53]. Pyridine Alkaloids, for example, can be produce by different plants species as *Nicotiana tabacum* L. or *Ricinus comunis* L. [54,55] and even pyridine ring can be part of other more complex alkaloids as berberine, an isoquinoline alkaloid which is the active constituent of plants as *Berberis* spp., [56], present in the vegetation series of the study area. There is also a possibility that pyridine becomes part of the environment through the feces and urine of animals medicated with sulfonamide antibiotics such as sulfapyridine [57,58]. In spite of the study area is located in the Sierra Nevada National Park, livestock grazing is frequent in areas adjacent to the highest villages when snow is not covering the vegetation, so we cannot dismiss this veterinary antibiotic as a possible source of pyridine in soil. Sulfapyridine whose structure includes a pyridine ring, can be degraded by laccase activity, releasing pyridine in one of its degradation steps [57].

Soil:water extract created with soil heated at 300 °C was the heating treatment with the highest number of peaks. This moderate heating allows the more complex and stable molecules present in UH samples, such as lignin compounds, to stay traceable, and, at the same time, it is expected that an increase in the yields of pyrolyzed material and a relative enrichment in n-alkylbenzenes, commonly observed in the pyrolysates of natural macromolecular materials [47]. Compounds, such as phenol, 2-methoxy-(guaiacol), were detected in both, UH and 300 °C samples, but with a higher relative abundance in the heated treatment. The higher recalcitrance of the lignin compared to the cellulose and the hemicellulose components of biomass [59] could explain this increase in the soil:water extract created with soil heated at 300 °C. At temperatures below 350 °C, the predominant reaction affecting lignin is dehydration [60], while other more labile compounds, such as cellulose, are expected to undergo important compositional changes above 270 °C [61]. According to Figure 3, aromatics and N-compounds are the main groups of molecules in soil heated at 300 °C. In fact, the major relative abundances detected in the 300 °C pyrogram were aromatic compounds (toluene, benzene, phenol), but also prominent peaks of furan, 2, 5-dimethyl, pyrocol, and other diketopyperazines were detected. Plant material as cellulose and pectin suffer slight changes at temperatures below 200 °C but above 300 °C char material are dominated by phenols, furans and aromatic hydrocarbons [6], as well as a temperature-induced denaturalization of proteins and polypeptides would be expected. This may explain the marked increase in aromatic compounds and diketopyperazines in the soil:water extract from the 300 °C soil heating. Nevertheless, the highest proportion of aromatic compounds was detected in soil:water extract created with soil heated at 450 °C, where more than 50% of the compounds detected presented aromaticity. Both, cellulose and pectin are expected to undergo increases in aromatic-C with temperatures above 350 °C [62,63]. The analysis of the volatile products of pectin pyrolysis showed that their composition changed with temperatures from mainly furans, pyranones, anhydrosugars, and 5-hydroxymethylfurfural at low temperatures to phenol, catechol, and substituted phenols at high temperatures [62], which could explain the increment in the proportion of aromatic compounds in soil:water extract made with soil heated at 450 °C compared with the one from a lower heating temperature. In a similar way, the pyrolysis of fatty acids present in unaltered samples could result in traces of aromatic compounds, such as toluene [64,65], which showed a marked increase in samples heated at 300 and 450 °C in our experiment. Sharman et al. [60] noticed that the nature of the substrate had only a small effect on the composition of char at high temperatures, for example, they found that the compositions of the lignin chars above 400 °C were similar to those of the chars from pectin or chlorogenic acid, showing in general, an increase in aromaticity. At temperatures approximately 400–500 °C, fused-ring systems could be formed from lignin, but the formation of PAHs depends, not only on the temperature, but also on other characteristics of the original material, such as aromaticity or surface area, which could be determinants [60]. The pyrograms of soil:water extract based on soil heated at higher temperatures (450–500 °C) showed an important peak of 9H-flueren-9-one, an oxygenated derivate of PAHs (O-PAH). Previous work has reported two- and three-ring PAHs detected in lignin char prepared at 350–400 °C, such as acenaphthylene, fluorene, phenanthrene, anthracene, and fluoranthene, while char prepared at 300 °C showed low concentrations of PAHs [60]. In our study, a small peak corresponding to naphtalene was detected in soil:water extract from soil heated at 300 °C but not at higher temperatures. The formation of these polycyclic compounds could also be due to the partial combustion of other substrates, such as cellulose. McGrath et al. [63] studied the formation of PAHs from the pyrolysis of cellulose over the temperature range of 300–650 °C, reporting detectable amounts of PAHs at and above 400 °C, while no PAHs were detected in the range of low temperatures (25–300 °C). The pyrolysis of furans, compounds that were detected in UH samples, could also produce PAHs, but would need temperatures of approximately 800–900 °C [66], which were not reached in our experiment. Due to the hydrophobic nature of PAHs [67,68], low concentration of these compounds in our experiment could be explained, since Py-GC/MS analysis was conducted on soil:water extract instead of the soil particles, so soluble organic compounds will be expected to be the majority.

Furthermore, the percentage of N-compounds was fairly similar in the soil:water extract created with UH and 300 °C soil samples (Figure 3), but the nature of the compounds was different. It is noteworthy the presence of several cyclic dipeptides in samples heated at 300 °C which are not present in any other sample (Figure 2). The thermal decomposition of proteins is characterized by systematic and random depolymerization reactions, and one of the classic products of this depolymerization are cyclic dipeptides, which are known as diketopiperazines [45,66]. Sharman et al. [69] reported that the amino acid proline was completely converted into volatile products at 300 °C, yielding approximately 80% of tar, of which the single major component was 2,5-diketopiracine (cyclo Pro-Pro). In our experiment, this compound was relevant, representing c. 7% of the compounds identified in the pyrogram of a 300 °C soil:water extract; also other cyclic dipeptides were identified as smaller peaks having proline as a common amino acid. Other sources of these cyclic compounds could be the pyrolysis of polypeptides, which can also undergo decarboxylation, deamination, and dehydration to dipeptides, followed by a second dehydration to yield a 2,5-diketopiperazine [6]. At higher temperatures (above 500 °C), polynuclear aromatic structures containing nitrogen (N-PACs) are also produced. In this experiment, higher temperatures of heating led to increased aromatic structures containing nitrogen as the nitrile group, as can be observed in the benzonitrile peak, which was the major compound in samples heated at 500 °C (Figure 2 and Figure 4). The presence of the nitrile group as the predominant N form in samples heated at higher temperatures could be related to polipeptides and proteins due to a further conversion of the cyclic dipeptides (diketopiperazines) that undergo further pyrolysis, decomposing by different mechanisms leading to the formation of nitrile or isocyanates, depending on the decomposition route [66]. De la Rosa et al. [47] proposed dehydration following C–N bond cleavages, favoring thermal oxidation, as a possible mechanism for nitrile formation.

Another prominent compound found in samples between 300 and 450 °C was the aromatic ketone acetophenone. A possible origin of this compound could be related to the pyrolysis of Amadori compounds that could have been formed by reacting glucose with amino acids and yielding ketones, aldehydes, pyridines, pyrazines, pyrroles, and carboxylic acids [70]. This compound has been reported in other studies in soil samples from burned Mediterranean forests [47].

The general trend with temperature appears to be the increase in aromaticity, with an important role of N-aromatic compounds, confirming previous observations related to heated soil with increases in the proportion of nitrogen relative to C through the development of thermally stable heterocyclic N [47].

### 3.4. Van Krevelen Diagrams

**Figure 5:**
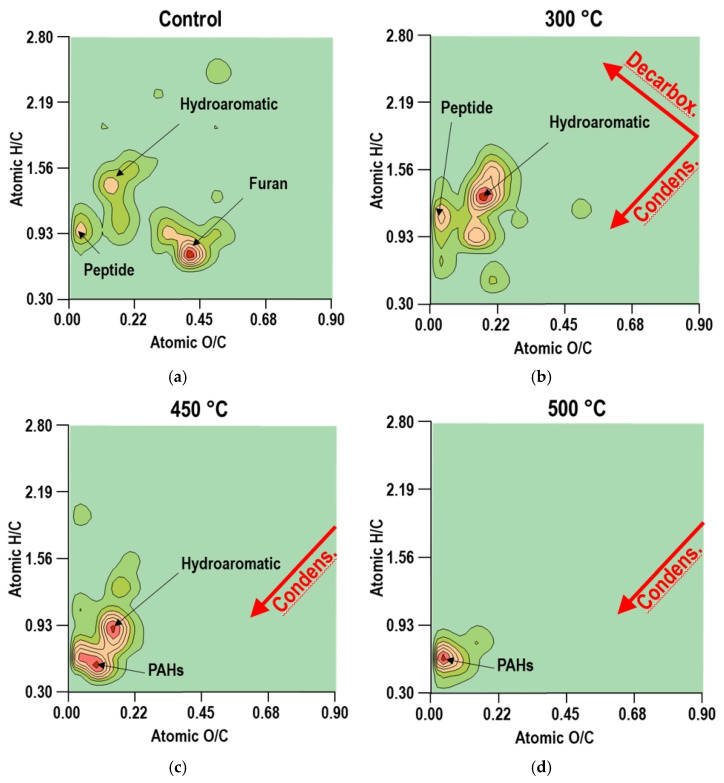
Van Krevelen diagrams representing the evolution of atomic ratios H/C and O/C at different temperatures of heating: unheated control (**a**), heated at 300 (**b**), 450 (**c**), and 500 °C (**d**).

Van Krevelen diagrams help us to condense the information explained in the previous section, showing the principal chemical reactions occurring with increasing temperatures. In general, we can observe that the general tendency is the diminution of both ratios, O/C and H/C, with increasing temperature (Figure 5). Similar results have been reported in the pyrolysis of single organic compounds, such as pectin, cellulose [62], or humic substances [71], identifying dehydration, decarboxylation, and decarbonylation as major reactions during pyrolysis. Medium-moderate heating in our experiment (300 °C) was characterized by decarboxylation and condensation reactions. If we compare the diagram of UH with the 300 °C one, we can observe a reduction in the O/C ratio that could be due to the release of CO_2_ due to the decarboxylation of a more labile compound. Rantuch and Chrebet [72] considered the dehydration of cellulose until 210 °C, and set 250 °C as the temperature at which glucose begins to break down. On the other hand, the diminution in H/C ratio due to condensation could be related to the loss of H2O from more stable compounds, such as lignin, that start the decarboxylation process at a higher temperature. Sharma et al. [60], in their characterization of chars from the pyrolysis of lignin, identified a more rapid loss of oxygen compared with hydrogen in samples above 350 °C and attributed it to decarboxylation processes, concluding that the predominant reaction was dehydration at low temperatures followed by dehydration and some decarboxylation at high temperatures.

In our study, at higher temperatures (450–500), the reduction in the O/C ratio was less intense than the H/C ratio if compared with the changes observed in samples heated at 300 °C compared to the UH samples. At these higher temperatures, more stable compounds are present, pointing to condensation as the predominant reaction. Heating at 450–500 ° C generates a higher proportion of compounds whose thermal stability is higher. Studies of the thermal stability of different organic compounds classified heterocydics, aromatics, and certain substituted aromatic compounds, perfluorinated ring compounds, and aromatic silanes as the most stable compounds [73]. Our results confirm previous observations, concluding that heat-induced dehydration and cyclization reactions lead to the accumulation of a large number of condensed structures, including heterocyclic nitrogen forms [5].

### 3.5. Abundance of Viable and Cultivable Micro-Organisms

Viable and cultivable microbial abundance was estimated by plate count methods, accounting for those micro-organisms able to grow in the different culture media prepared (Table 1) and inoculated with the same unaltered soil.

#### 3.5.1. General Heating Effect

**Figure 6:**
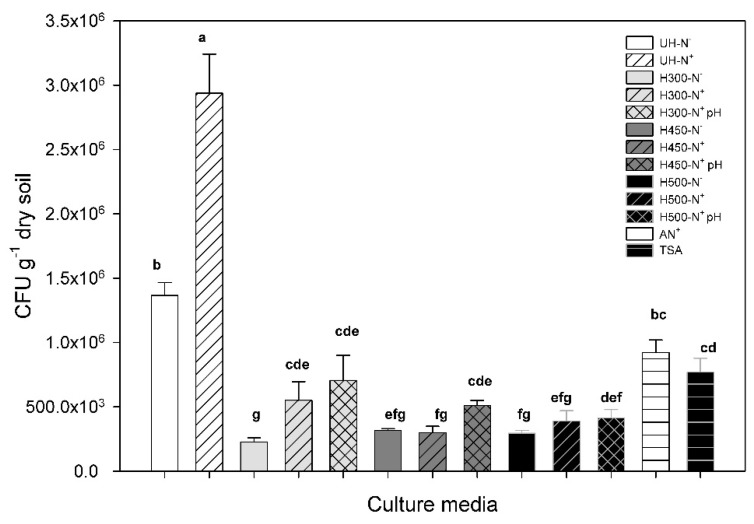
Mean value (± SE) of CFUs counted in each culture media (Table 1). Different letters indicate significant differences among treatments according to Tukey’s test (α = 0.05). The codes of the different soil:water extract media are described in Table 1. AN^+^ media was created only with nutrient solution as control of the application of nutrients amendment. TSA (Tryptic Soy Agar) was used as a reference for typical culture media to isolate soil micro-organisms.

There was a marked difference between the CFU accounted for in culture media prepared with unaltered soil extract compared with those created with heated soils, despite the heating temperature (Figure 6). Treatments without nutrient and pH compensation show the influence of several factors at the same time, hindering the distinction between the effect caused by the decrease in nutrients, the variation in the type of compounds, or the changes in pH.

#### 3.5.2. Compensation of Nutrient Weakening Induced by Heating

Nutrient addition led to a microbial increase compared with unamended media in both heated and unheated soil-based culture media. The N^+^-treatment provided a minimum for growth, so the results should be compared with the media AN^+^ to discern the number of micro-organisms that are able to grow under these nutrient conditions. For the media prepared with unheated soil extract and amended with nutrients, we found the highest CFU abundance, doubling the amount of CFU accounted for in unheated and unamended soil-based culture media, in TSA, or in N^+^-Agar. This conspicuous rise in the microbial proliferation observed with the addition of nutrients could be due to a possible growth limitation present in UH-N-media caused by the scarcity of some essential nutrients, which could be preventing the maximum exploitation of the nutritional soil resources. Considering that the nutrient amendment was rich in N, and taking into account the C to N ratio shown in the soil characterization (C/N = 31.4), it is probable that the N enrichment could stimulate microbial proliferation. Nitrogen is an essential element that can limit the growth of living organisms across a wide range of ecosystems [74], but general results in the bibliography evidence that microbes are most commonly limited by carbon availability, even in soils with high OM content [75,76]. However, there is evidence that strongly suggests that microbes in natural soils are sometimes N-limited, although the results obtained are not easily interpretable [77]. Some studies reported no effects or even a negative effect on N supply even in a C-rich environment [76,77,78], suggesting that N was not a limiting factor for microbial growth, but rather had a synergistic effect when C and N were added together [79]. This fact could explain the difference in the CFU counted in UH-N^+^ media compared with UN-N-, since the nutrient amendment used in our experiment not only provided an N-source, but supplied P and C sources as well.

Media prepared with heated soil and nutrient compensation showed higher CFU abundance than the unamended ones at 300-N^+^ and 500-N^+^, but the abundance was always below that in culture media created with unheated soil and even in AN^+^ media. The culture media prepared with soil heated at 450 °C did not show significant differences in the nutrient compensation, evidencing the presence of other factors limiting the microbial growth.

#### 3.5.3. Compensation of pH Rise Induced by Heating

The results obtained with the culture media containing nutrients and pH compensation point to organic matter quality as the most possible factor influencing the CFU number. This, together with the result mentioned before, allowed the isolation of the magnitude in which these parameters (pH, nutrient availability, and possible changes in the quality of the organic compounds) condition the proliferation of viable and cultivable micro-organisms in the unaltered area. The pH compensation showed a positive effect on the culture media created with heated soil at 300 and 450 °C, although the differences were significant only at the highest temperature, where the fire-induced pH change was more accentuated, changing from 5.9 in the original soil to 7.3 in the heated one. Bárcenas-Moreno et al. [19] in their research about functional implications of the pH-trait distribution of the bacterial community, found that inoculum communities derived from low pH had lower than optimal pH-tolerance when inoculated in high soil pH environments. Apart from the natural adaptation of the original microbial community that certainly could be affecting CFU proliferation, fire-induced pH changes could affect some enzymatic activities essential for degrading compounds that could be limiting microbial growth in this treatment, such as PAHs. In fact, fungal population appears to play an important role in PAH degradation, with an important role in its lignolytic activities as laccase [80,81]. The pH effect on PAHs’ degradation appears to depend on different factors, such as organism species or the PAH compound. Vipotnik et al. [81] evaluated the effect of soil pH on polycyclic aromatic hydrocarbons degradation and determined that fluorene, pyrene, and benzo[a]pyrene achieved higher degradation rates in soil at pH 5 and established that c. 85–90% of the PAHs were degraded by fungal remediation. On the other hand, Pawar [82] established pH 7.5 to be the most suitable for the degradation of phenanthrene, anthracene, fluoranthene, and pyrene, but when using a bacterial consortium instead of fungi, the optimal soil pH for PAH degradation seems to be highly dependent on the microbial community composition. The characterization of fungi with laccase activity in our study area was conducted by Bárcenas-Moreno et al. [83], who found that 30% of isolated fungi express laccase activity in unaltered areas (UHs) and reported an increase in this percentage to 40% in a nearby area affected by a wildfire 6 years earlier. The effect of this wildfire on pH in the study area was a slight decrease that was still present 20 months after the fire [25] and a marked increase in the fungi to bacteria ratio based on plate count results. These results could support the hypothesis that pH re-establishment in culture media based on soil heated at 450 °C could promote a greater proliferation of CFU through the growth of micro-organisms able to degrade toxic compounds present in the sample, such as fluorenone, whose degradation could be limited at a higher pH. The 9H-fluerenone can be degraded by bacterial species, such as *Pseudomonas* sp., *Brevibacterium* sp., and also *Arthrobacter* sp., by angular deoxygenation [84,85], but also by fungal species. Different strains of ascomycetes, basidiomycetes, and deuteromycetes have also been reported as fluorene degraders [86], with an important role for white-root fungi, such as *Armillaria* sp., which can degrade 9-fluorenone by oxidative decarboxylation [87] and express optimal growth in acid media [88].

It should be highlighted that the pH compensation did not evidence any effect at 500 °C, despite this heating treatment showing a higher heating-induced pH rise compared to the original soil pH. This may reflect the marked effect of fire-induced organic matter composition at this temperature.

#### 3.5.4. Effect of Qualitative Changes in Organic Matter

Analyzing the results, and taking into account that every culture medium was inoculated with the same soil inoculum, there seems to be a clear negative effect of heated soil extract on microbial proliferation and that this is not entirely compensated with nutrient addition nor by pH re-establishment, especially at higher heating temperatures (450–500 °C).

According to Py-GM/MS results, the increase in aromatic compounds, such as toluene, phenol, related compounds, or furans at 300 °C could explain why the increase shown with amendments was not enough to reach the CFU values measured at UH or AN+, in spite of the fact that DOC and pH did not experiment on important heating-induced changes compared to the control. Previous studies have attributed the toxicity of DOC from biochar to the presence of phenolic compounds or low-MW organic acids [89,90] and also to the increase in the amount of organics containing <6 O atoms and 1 N atom per compound when the temperature of production is >300 °C [90]. The presence of an important proportion of acetophenone in the culture media created with soil heated at 300 and 450 °C could be another component limiting microbial proliferation. The toxicity of acetophenone on micro-organisms has been previously studied, reporting fungal and yeast growth inhibition [91,92] and also the existence of different micro-organisms able to degrade it by different metabolic pathways [93].

The presence of PAHs, mainly in the 450 °C treatment, and their microbial inhibition have been previously discussed regarding pH heating-induced changes and the consequences of pH re-establishment, which in our study appeared to have a positive effect on CFU proliferation. Although PAHs in soil can cause toxicity to micro-organisms, the microbial community is able to degrade these compounds, so their toxic effect in our experiment could be modulated by the microbial community composition and the fire-induced changes to those factors controlling the proliferation of PAH degraders. In contrast, the pH re-establishment on culture media created with soil:water extract from 500 °C heating did not produce any change in CFU proliferation. In this treatment, CFU only showed a slight increase with nutrient amendment. Due to the low number of compounds present in this treatment, CFU counted on the culture 500-N-, showed micro-organisms not only resistant to the presence of compounds, such as benzonitrile, but also capable of degrading it as a only substrate to growth. Several bacterial strains have been identified to be resistant to benzonitrile-related compounds, such as Azotobacter chrooccum [94], and also to be capable of growing on mineral medium containing benzonitrile as a sole source of carbon and nitrogen with maximum biodegradation at different soil pH [95]. In our study, the increase in CFU in 500-N^+^ compared to 500-N- could be due to the supply of other carbon and nitrogen sources that could allow the appearance of micro-organisms with the capacity to grow in the presence of benzonitrile, but not be able to use it as a sole source to grow. In addition, Ajane and Dharmadhikari [95] found that the presence of casein as a nitrogen source and fructose as a carbon source increased benzonitrile hydrolysis, so the nutrient amendment in our study could increase benzonitrile degradation, allowing the proliferation of micro-organisms less tolerant to the presence of benzonitrile.

Analyzing microbial and organic matter composition results together, we can connect the inhibition of microbial proliferation to both the diminution of organic compounds available to decompose and the rise of the relative abundance of fire-induced compounds, such as PAH and NCC, not only due to their recalcitrant nature but also to their toxic and inhibitory effect on soil micro-organisms.

## 4. Conclusions

This study brings to light that the magnitude in which fire-induced changes on soil pH or on organic compounds determines microbial response largely depends on the heating temperature applied. This fact entails an important finding for a better understanding of other physicochemical parameters after fire and for planning possible strategies for the ecosystem’s recovery, based on severity index.

We observe that samples heated at 300 °C showed insignificant changes in pH or DOC but showed an increase in the proportion of aromatic compounds together with a reduction in polysaccharides and the presence of phenols from lignin, turning it into a rather diverse but recalcitrant growth media. This finding can help us to explain the variability of results found on microbial parameters in low-moderate severity fire and should make us pay more attention to fires that apparently could be classified as low-severity fire but, that in turn, could exert an important delay in microbial recovery. The toxic and recalcitrant nature of compounds identified in samples heated at 450 °C confer to pH fire-induced changes a determinant role on microbial proliferation, which will be condition by the original soil pH and microbial community composition. Thus, in the future we will be able to better interpret the implications of pH fire-induced changes on microbial response and ecosystem recovery, taking into account not only the change but also the original pH which determine microbial community composition.

Changes in quantity and quality organic matter seems to be the most important factors conditioning microbial growth in samples heated at 500 °C, in spite of the marked increase experimented by pH. In this situation, the original microbial community composition will play a determinant role managing negative effect of the organic compound scarcity, recalcitrance, and toxicity.

Future research, including the whole of the microbial community and a wider range of fire temperatures are needed for a better understanding of microbial response and its role in ecosystem recovery, adding other physicochemical fundamental parameters related to soil functioning.

## Figures and Tables

**Table 1:** Description of different culture media prepared with soil:water extract.

Treatment	Heating Temperature (°C)	Nutrient Addition	pH Rectification	Final pH
UH N^−^	Unheated	NO	NO	6.3
UH N^+^	Unheated	YES	NO	6.1
H300 N^−^	300	NO	NO	7
H300 N^+^	300	YES	NO	7
H300 N^+^ pH	300	YES	YES	6.2
H450 N^−^	450	NO	NO	7.3
H450 N^+^	450	YES	NO	7.3
H450 N^+^ pH	450	YES	YES	6.1
H500 N^−^	500	NO	NO	7.4
H500 N^+^	500	YES	NO	7.4
H500 N^+^ pH	500	YES	YES	6.1

**Table 2:** Results of soil characterization of the samples collected in the unaltered high mountain shrubs ecosystem before laboratory heating and the preparation of different culture media (mean value ± standard error).

Soil Parameters	Mean ± Standard Error
Soil organic carbon (g C kg^−1^ dry soil)	70.36 ± 1.25
Kjeldahl Nitrogen (g N kg^−1^ dry soil)	2.31 ± 0.27
C:N Ratio	31.4 ± 0.4
Electrical conductivity (1:2.5, μS cm^−1^)	332.5 ± 5.5
Texture	Sandy loam
Microbial Biomass-C (mgC kg^−1^ dry soil)	275.79 ± 26.34
Respiration rate (μg CO_2_ h^−1^ g^−1^ dry soil)	9.95 ± 0.55
Bacterial growth (Leu pmol h^−1^ g^−1^ dry soil)	100.96 ± 13.1
Bacterial PLFAs (nmol g^−1^ drysoil)	288.69 ± 32.19
Fungal PLFAs (nmol g^−1^ dry soil)	73.799 ± 8.54

**Table 3:** Different organic compounds identified in the different samples. Cmp = Reference number for the compounds used to cite them in the text; RT = Retention Time; m/z: Main diagnostic fragments; MW = Molecular weight.

Cmp	RT	UH	300	450	500	Library/ID	*m*/*z*	MW
**1**	2.21	0.0	0.0	4.3	0.0	2-Butene, 2,3-dimethyl-	69, 84	84
**2**	2.28	8.6	5.6	0.0	0.0	Pyridine	52, 79	79
**3**	2.39	1.7	9.5	6.5	0.0	Benzene, methyl-(Toluene)	91, 92	92
**4**	2.46	4.1	0.0	0.0	0.0	Formamide, N-methyl-	59	59
**5**	2.47	0.0	6.8	2.4	0.0	Cyclopentanone	55, 84	84
**6**	2.55	5.8	0.0	0.0	0.0	2H-Pyran, 3,4-dihydro-	55, 84	84
**7**	2.61	7.4	0.0	0.0	0.0	3-Furancarboxaldehyde	67, 94	94
**8**	2.75	0.0	8.0	4.2	0.0	Furan, 2,5-dimethyl-	53, 81, 96	96
**9**	2.76	11.1	0.0	0.0	0.0	2-Furancarboxaldehyde (Furfural)	67, 94	94
**10**	2.98	0.0	6.6	0.0	0.0	Benzene, 1,3-dimethyl-	91, 106	106
**11**	3.00	1.7	0.0	0.0	0.0	Pyridine, 4-methyl-	66, 93	93
**12**	3.13	2.0	0.0	0.0	0.0	4-Cyclopentene-1, 3-dione	54, 68, 96	96
**13**	3.19	0.0	3.0	3.3	3.5	Cyclohexanone	55, 69, 98	98
**14**	3.20	2.2	0.0	0.0	0.0	Styrene	78, 104	104
**15**	3.28	0.0	2.5	0.0	0.0	2-Cyclopenten, 1-one, 2-methyl-	53, 67, 96	96
**16**	3.35	1.6	0.0	0.0	0.0	2,3,4-Trimethylfuran	81, 95, 110	110
**17**	3.39	4.8	0.0	0.0	0.0	2(5H)-Furanone	55, 84	84
**18**	3.39	0.0	2.2	0.0	0.0	1H-Pyrrole-1-ethyl-	53, 80, 95	95
**19**	3.53	0.0	2.1	0.0	0.0	2-Cyclopentene-1-one, 2,3-dimethyl	67, 95, 110	110
**20**	3.77	0.0	0.0	12.9	10.5	Benzaldehyde	51, 77, 106	106
**21**	3.79	8.6	0.0	0.0	0.0	2-Furancarboxaldehyde, 5-methyl	53, 110	110
**22**	3.85	3.8	6.6	3.7	0.0	Phenol	66, 94	94
**23**	3.92	0.0	0.0	0.7	0.0	1H-Pyrrole-2, 5-dione	54, 69, 97	97
**24**	4.01	2.0	4.0	23.4	62.2	Benzonitrile	76, 103	103
**25**	4.15	2.3	0.0	0.0	0.0	Pyridine, 3-methoxi	66, 94, 109	109
**26**	4.23	2.8	0.0	0.0	0.0	2-Acetylpyrrole	66, 94, 109	109
**27**	4.50	0.0	1.8	0.0	0.0	2-Cyclopenten-1-one, 2,3-dimethyl-	67, 95, 110	110
**28**	4.60	0.0	1.6	0.0	0.0	Phenol, 2-methyl-	77, 107, 108	108
**29**	4.60	2.0	0.0	0.0	0.0	Benzaldehyde, 2 hydroxy-	65, 93, 122	122
**30**	4.80	0.0	7.8	13.4	0.0	Acetophenone	77, 105, 120	120
**31**	4.84	2.5	0.0	0.0	0.0	Benzamine, 2-methoxy	80, 108, 123	123
**32**	5.00	0.0	0.0	1.6	0.0	2, 5-Pyrrolidinedione, 1-methyl-	56, 113	113
**33**	5.07	1.7	2.3	0.0	0.0	Phenol, 2-methoxy-	81, 109, 124	124
**34**	5.38	0.0	2.9	0.0	0.0	Succinimide	56, 99	99
**35**	5.49	3.2	0.0	0.0	0.0	2-Cyclohexen-1-one, 3,5-dimethyl-	54, 82, 124	124
**36**	5.64	1.3	1.4	0.0	0.0	Benzyl nitrile	90, 117	117
**37**	5.76	2.1	0.0	0.0	0.0	2,4(3H, 5H)-Furandione, 3-ethyl	55, 70, 100, 128	128
**38**	5.84	0.0	3.0	0.0	0.0	Phenol, 3,4-dimethyl-	77, 91, 107, 122	122
**39**	5.85	2.1	0.0	0.0	0.0	Pentane, 1,5-dimethoxy-	71, 100, 117,128	128
**40**	6.17	2.6	0.0	0.0	0.0	6-(tert-butyl)-2(1H)-pyridone	136, 151	151
**41**	6.22	0.0	2.6	0.0	0.0	Naphtalene	102, 128	128
**42**	6.24	1.8	0.0	0.0	0.0	Cyclohexanol, 2-(1-methylethyl)-	57, 69, 85, 98, 144	144
**43**	6.35	1.9	0.0	0.0	0.0	Furan, tetrahydro-2, 5-dimethoxy-	72, 101, 131	131
**44**	6.40	0.0	1.7	0.0	0.0	1,4:3,6-Dianhydro-.alpha.-d-glucopyranose	57, 69, 85, 98, 144	144
**45**	6.55	2.6	0.0	0.0	0.0	Benzenamine, 3-ethoxy-	80, 109, 137	137
**46**	6.75	1.0	0.0	0.0	0.0	Methenamine	112, 140	140
**47**	6.86	0.3	0.0	0.0	0.0	Benzenepropanenitrile	91, 131	131
**48**	6.91	1.3	0.0	0.0	0.0	Gly-Val	85, 114, 141, 156	156
**49**	7.06	0.5	0.0	0.0	0.0	Caprolactam	55, 85, 113	113
**50**	7.20	0.0	0.0	0.0	13.5	Dicyanobenzene	50, 75, 101, 128	128
**51**	7.48	1.0	0.0	0.0	0.0	Benzoic acid, 2,5-dihydroxy	80, 108, 136, 154	154
**52**	8.22	0.0	0.0	1.0	0.0	1(3H)-Isobenzofuranone	77, 105, 134	134
**53**	8.30	0.8	0.0	0.0	0.0	Pro-Gly	83, 98, 111, 154	154
**54**	8.51	0.0	0.0	0.4	0.0	1-Tetradecene	55, 196	196
**55**	8.59	0.0	0.0	2.9	0.0	Byphenyl	76, 154	154
**56**	9.09	0.0	0.5	0.0	0.0	1H-Isoindole-1, 3(2H)-dione, 2-methyl-	76, 104, 117, 132, 161	161
**57**	10.27	0.0	0.0	2.7	2.2	Dibenzofuran	84, 193, 168	168
**58**	11.55	0.0	0.0	0.7	0.0	Benzophenone	77, 105, 182	182
**59**	12.27	0.0	0.5	0.0	0.0	Cyclo (Pro-Ala)	70, 97, 125, 168	168
**60**	12.43	0.0	5.0	0.0	0.0	Pyrocoll	65, 93, 130, 186	186
**61**	12.66	0.0	0.9	0.0	0.0	Cyclo (Pro-Ala)	70, 97, 125, 168	168
**62**	12.80	0.0	0.0	15.9	8.1	9H-Fluoren-9-one	76, 152, 180	180
**63**	12.89	0.0	1.0	0.0	0.0	Cyclo (Pro-Gly)	70, 83, 98, 111, 154	154
**64**	13.34	0.5	0.0	0.0	0.0	Phenol, 4,4′-methylenebis-	107, 183, 200	200
**65**	13.50	0.0	1.2	0.0	0.0	Cyclo (Pro-Ala)-(CN3H4)	70, 125, 154	154
**66**	13.77	0.0	2.1	0.0	0.0	Cyclo(Pro-Val)	70, 72, 125, 154, 196	196
**67**	14.79	0.0	6.7	0.0	0.0	Cyclo(Pro-Pro)	70, 96, 138, 166, 194	194
**68**	15.35	0.5	0.0	0.0	0.0	Hex-1-ene, 2,5-diphenyl-	91, 118, 236	236

## Data Availability

Complete data sets are available upon request.
